# Continuing Professional Development ‐ Radiation Therapy

**DOI:** 10.1002/jmrs.826

**Published:** 2024-09-20

Maximise your CPD by reading the following selected article and answer the five questions. Please remember to self‐claim your CPD and retain your supporting evidence. Answers will be available via the QR code and published in JMRS – Volume 72, Issue 4, December 2025.

## Radiation Therapy – Original Article

### Comparing immobilisation devices in gynaecological external beam radiotherapy: Improving inter‐fraction reproducibility of pelvic tilt

Prasad S , Bell LJ , Zwan B , Ko F , Blackwell T , Connell K , Stanton C , Shepherd M , Atyeo J , Stevens M , Morgia M. (2024). J Med Radiat Sci 10.1002/jmrs.804 PMC1163837238894671

1. How much did re‐imaging for pelvic tilt decrease by using the BodyFIX system?
  - 19.4%
  - 32.6%
  - 50.0%
  - 73.5%
2. How many patients stabilised with the BodyFIX system required a planning CT rescan during treatment due to pelvic tilt issues?
  - 0
  - 1
  - 2
  - 3
3. In the survey, what percentage of departments reported seeing pelvic tilt on pre‐treatment imaging?
  - 30%
  - 50%
  - 55%
  - 82%
4. What was the median pelvic tilt difference for patients using the BodyFIX system?
  - 0°
  - 1°
  −2°
  - 2°
5. When during the treatment course did the patients who were stabilised using the BodyFIX system have variations in pelvic tilt of ≥5°?
  - Fraction 1–5
  - Fraction 6–25
  - All fractions
  - None of the above

## Answers

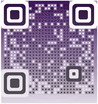

Scan this QR code to find the answers.
